# Biceps femoris late latency responses and the “notching sign” in spasticity

**DOI:** 10.1186/s12984-015-0084-7

**Published:** 2015-10-20

**Authors:** Mehmet Gürbüz, Süleyman Bilgin, Yalçın Albayrak, Ferah Kızılay, Hilmi Uysal

**Affiliations:** Department of Neurology and Neurophysiology, Akdeniz University Faculty of Medicine, B Block Level 2, Dumlupınar Bulvarı, 07070 Antalya, Turkey; Akdeniz University Faculty of Electric and Electronic Engineering, 07070 Antalya, Turkey; Sakarya University Institute of Natural Sciences, 54000 Adapazarı, Turkey

**Keywords:** Ashworth scale, Long latency responses, Medium latency responses, Notching sign, Patellar pendulum, Patellar T reflex, Spasticity

## Abstract

**Background:**

Spasticity is a motor impairment due to lesions in the brain and spinal cord. Despite being a well-known problem, difficulties remain in the assessment of the condition. The electrophysiological and kinesiological characteristics of the patellar pendulum changes during the movement triggered by the patellar T reflex could be used to assess spasticity.

**Methods:**

Features of the patellar pendulum during the patellar T reflex were considered using a goniometric approach in spastic patients evaluated with the Ashworth scale. Medium and late latency responses in the rectus and biceps femoris muscles were examined electrophysiologically. For each pendulum, the maximum angle extension during an oscillation of the knee joint, maximal extension time, angular velocities of extensions of the knee joint and frequency of motion due to the patellar reflex were calculated. The damping of the amplitude in the pendulum was calculated.

**Results:**

The spasticity group consisted of 65 patients (38 males and 27 females) with a mean age of 47.6 ± 14.0 years. The normal control group consisted of 25 individuals (19 males and six females) with a mean age of 32.1 ± 10 years. The biceps and rectus femoris long latency late responses were not observed in the normal cases. The biceps femoris medium latency response was observed only in 24 % of healthy individuals; conversely, late responses were observed in 84 % of patients. Activation of the antagonist muscles at a certain level of spasticity created a notching phenomenon**.** Amplitude of the reflex response and mean angular velocity of the first oscillation present in a dichotomic nature in the spasticity groups. Frequency of the first pendular oscillation increased with the increase of the Ashworth scale, while the damping ratio decreased with increasing scale. The Ashworth scale showed a correlation with the damping ratio. The damping ratio strongly distinguished the spastic subgroups and showed a strong negative correlation with the Ashworth scale.

**Conclusions:**

The Ashworth scale presents a good correlation with kinesiological parameters, but it is only possible to differentiate normal and spastic cases with electrophysiologic parameters. Furthermore, the notching phenomenon could be evaluated as a determinant of spasticity.

## Background

Although spasticity is a well-known motor disorder in clinical neurology, problems remain in the definition, assessment and measurement of this disorder [1, 2]. Knowledge of the mechanisms of spasticity has been derived almost entirely from electrophysiological findings [3–5]. However, agreement between clinical and electrophysiological assessments is not as expected [6]. A definitive electrophysiological method compatible with clinical findings, which could be used to assess spasticity, has not yet been developed [7, 8]. As it is a maladaptive complex motor response to a lesion of the central nerve system, it is not a simple issue to understand the output.

Because spasticity is defined as the velocity-dependent resistance of the joint against passive motion, isokinetic studies have been used during assessment [9]. The current approach is to use a combination of neurophysiological and biomechanical methods in the assessment of spasticity during not only passive motion but also during voluntary movements [4, 10]. However, electromyographic and dynamometric findings of patients with high Ashworth scales shows very little compliance with spasticity [2, 11].

If the patellar tendon is stimulated using a tendon hammer while the foot is hanging free, there is a series of to and fro movements of the foot and leg before the limb finally comes to rest. This response is normally prevented by the after-shortening of the quadriceps femoris. Late latency responses (LLR) and pendular deformations during the patellar T reflex-triggered patellar pendulum were mentioned in early studies [12–15]. However, it is surprising that there are no systematic studies exploring the relationship between late responses and AS.

The aim of this study is to investigate the interaction between muscle activity and the angular displacement in spasticity via the patellar T reflex and pendulum to improve clinical scales of spasticity. The electrophysiological and kinesiological changes during the patellar pendulum triggered by the patellar T reflex in spasticity should present clues for assessing the pathological state.

## Materials and methods

Patients with spasticity and control subjects with normal neurologic findings were included in the study. The study was reviewed by the local ethics committee and complies with the Helsinki Clinical Research principles.

Spasticity patients were divided into 4 groups (Ashworth 1, 2, 3, and 4) according to the AS rating of the knee extensors [16]. In the spasticity group, patients with cerebral palsy, patients with lower motor neuron lesions, and those with cerebellar findings were excluded from the study. Measurements were taken before the patients were put on antispastic medication or before taking the next dose in the case of patients already on medication. The control group was composed of 25 volunteers showing no signs of neuromuscular disorder following neurological examination.

Individuals were seated in the most comfortable position on the examination couch, leaning back with feet high above the ground with supports made of sponges placed under their arms. For clinical assessment of spasticity, the knee joint was moved from maximum extension to maximum flexion.

To assess kinesiologic features, a DATALOG Type No. MWW8 Bluetooth® portable EMG + goniometer device produced by Biometrix was used. The proximal part of the sensor (Biometrix® twin axis goniometer, SG150) was inserted laterally into the knee, and the second part was inserted below the knee; the parts were then held in place with adhesive tape. Information on the angular velocity of the motion, the frequency and amount of motion, that is, how far the ankle had moved from its initial position, and the extent of the sequential movements (if any) were analyzed using the goniometer device. The sampling rate was 50 Hz. In addition to angular movement, muscle activity was also recorded through surface electromyography (EMG) electrodes placed on Rf and Bf muscles. EMG recordings were made using a Synergy EMG device. The lower frequency limit was 50Hz and the upper frequency limit was 10 kHz, the sweep speed was 200 msec/div., and the sampling rate was 10 kHz during the recording. The amplitude was recorded as the peak to peak of the response (μV). The latencies were measured in msec from the beginning of the response from the baseline. The initial knee joint motion, which was caused by the patellar tendon reflex, followed the occurrence of the muscle action potential. This motion was recorded along with the tendon reflex using a motion-sensitive sensor (accelerometer, CareFusion®) on the sagittal axis. This was placed between the 2nd and 3rd metatarsophalangeal joints on the foot. The lower and upper frequency limits were 0.1 Hz-50 Hz during the recording. The same sweep time was used as with the simultaneously recorded EMGs.

The lower extremity was placed in a position perpendicular to the ground, and the knee joint was left in a free state. The starting angle of the knee joint represented “zero.”

The patellar reflex was stimulated with an electronically triggered reflex hammer (084C001 – Nicolet – Viasys Tendon Hammer –Synergy). The point of best response was determined at 8 cm distal perpendicular to the patellar tendon and the researcher aimed to tap this point. Assessment was carried out using the parameter with the shortest latency and highest amplitude among the electrophysiological parameters belonging to ten patellar reflexes.

The three muscle activities, referred to as M1, M2 and M3, are defined as relating to short latency reflex (SLR), medium latency reflex (MLR), and long latency reflex (LLR), respectively [17]. Late potentials, which occurred after the M response in the Rf following the patellar T reflex, were termed as M2 and M3 in the Bf muscle. In this study, the response observed in the Rf muscle was a counterpart of M1 and was defined as the T reflex. SLR observed in the Bf muscle may be confused with the volume conduction of the response relating to the T reflex., Responses compatible with T reflex latency in Bf were not included in the assessment to account for other possibilities such as irradiation of reflexes [18]. Responses observed in Bf up to 65 msec were defined as M2, while responses observed after 65 msec that did not enter into the second oscillation were termed as M3. Latencies were measured according to the triggering time of the reflex hammer. The time measured by the accelerometer was subtracted from the recorded latency, and the corrected Bf-M2 and M3 latencies were calculated. Responses that followed the T reflex within the first oscillation in the Rf were considered as Rf-late responses. Medium and late latency responses were only considered if the motion of the joint continued in the same direction.

**Fig. 1:**
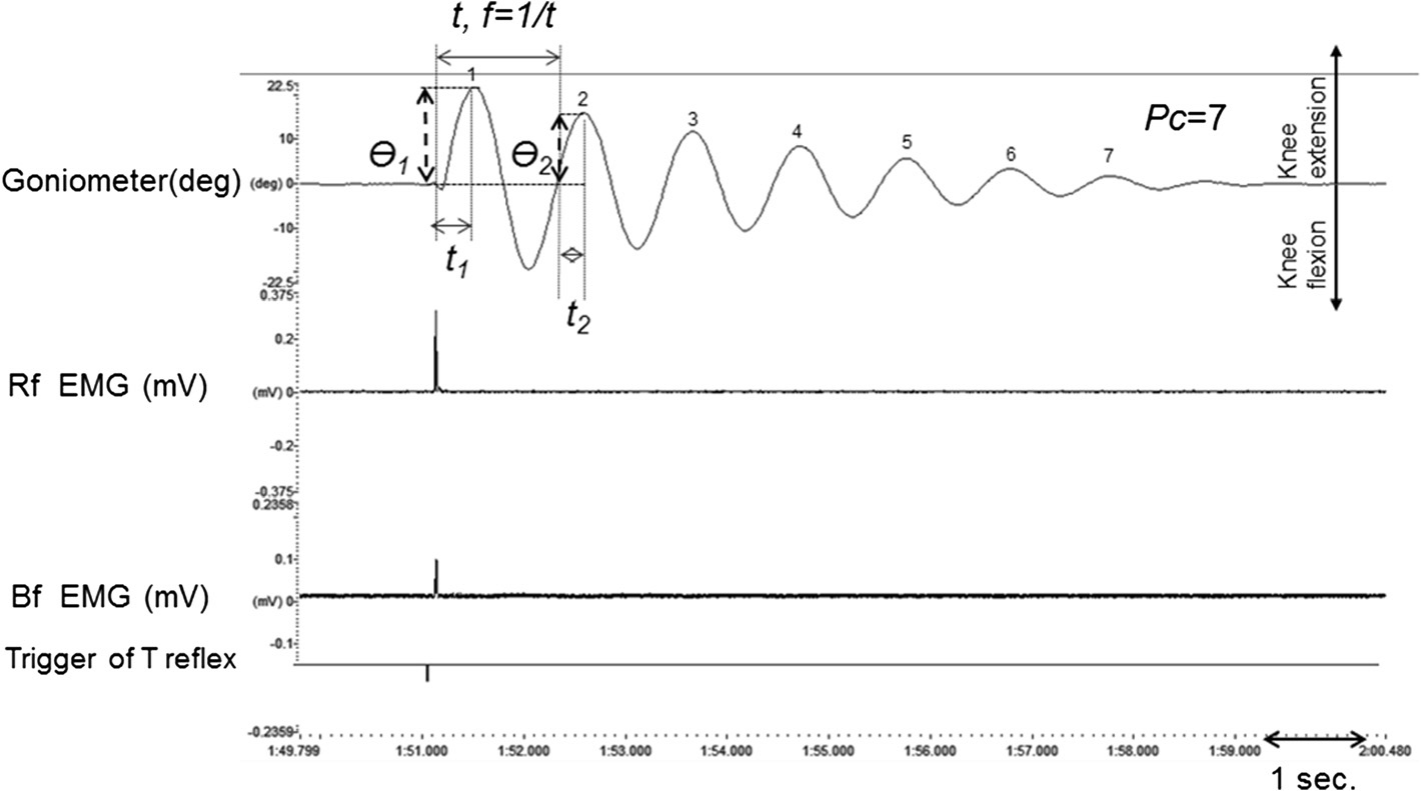
A pendulum triggered by the patellar T reflex -Normal case. The top trace shows the pendulum triggered by the patellar T reflex, recorded using a goniometer. The knee angle is 90°. The pendulum starts with a knee extension and ends with a seven beat (Pc) oscillation. The damping ratio (DR) is calculated as the ratio of Ө_2_ / Ө_1_. The oscillation frequency (f) has been calculated by the duration of the first beat (t) as 1 / t. The second row of the traces is rectified surface EMG recorded from the rectus femoris muscle. In the third row, rectified EMG activity of the biceps femoris muscle is displayed. The bottom trace indicates that the patellar T reflex has been triggered. The scale is shown on the vertical line. The upper scale is the angle in degrees, while the middle and lower traces scales are shown in mV amplitude. The sweep speed is shown on the bottom right

The following values were measured individually for the three pendulums with the greatest amplitudes, and the mean values were recorded. The maximum extension angle (Ɵ_*1*_, degree) during the first oscillation of the knee joint and maximum extension angle in the second oscillation (Ɵ_*2*_, degree) caused by the patellar T reflex were measured separately by electrogoniometer. The time taken to reach the maximal extension (*t*_*1*_*, t*_*2*_, second) was calculated for both oscillations. The mean angular velocities of the maximal extensions of the knee joint motion caused by the patellar T reflex were measured for the first and second oscillations (*ω*_*1*_ = Ɵ_1_ / t_1_, degree/second), and the frequency (*f,* Hz) of knee joint motion caused by the patellar reflex was calculated using the duration of a cycle. The ratio of the first and second beat amplitudes of the each pendulum was used to calculate the Damping Ratio (*DR*) (Ɵ_*2*_/ Ɵ_*1*_) (Fig. 1). The number of the pendulum (*Pc*, n) was determined using a goniometer and was defined using the same terms as used previously.

Statistical analysis was carried out using SPSS 18.0 software. *P* values < 0.05 were considered statistically significant. Mean values were expressed with standard deviations (sd) and min-max values. In the inter-group comparisons, the Kruskal-Wallis test was used to analyze the significance of differences between the electrophysiological and kinesiological parameters. The Mann–Whitney *U* test was used to define the group from which the significances originated. Correlations were evaluated using Spearman’s rho.

## Results

Our patient group consisted of 65 patients with spasticity caused by upper motor neuron lesions. The patients were aged between 18 and 75 years with a mean age of 47.6 ± 14.0 years. The control group consisted of 25 individuals with 19 males and six females aged between 20 and 55 years with a mean age of 32.1 ± 10 years.

Of the spasticity patients, 38 were male and 27 were female. The duration of the disease that caused spastic hypertonia was a minimum 1.5 months and a maximum 30 years, with a mean value of 8.6 ± 8.6 years. Lower extremity findings were bilateral in 40 of 65 cases, while upper motor neuron findings were unilateral in 25 cases, and there was no involvement in the other extremity. In 24 of 36 patients with spasticity due to cerebral lesion, both spastic and normal extremities were assessed separately, whereas in the patients with spastic paraparesis due to spinal lesions, recordings were carried out from the side with prominent spasticity. The Ashworth 1 group consisted of 18 individuals, the Ashworth 2 group consisted of 23 individuals, the Ashworth 3 group consisted of 18 individuals, and the Ashworth 4 group consisted of six individuals. The mean height was found to be 165.9 ± 9.2 cm in the spastic group and 172.3 ± 8.5 cm in the control group.

### Electrophysiological and kinesiological features of the control group

The latency of the patellar T reflex was measured as 17.9 ± 1.9 msec, and the amplitude was 6404 ± 2860 μV. The beginning time of the joint movement was measured with an accelerometer as 51.5 ± 9.9 msec. The Bf-M2 response was observed in 6 cases, and the observation rate was 24 %. The corrected latency of the Bf- M2 response was 33.0 ± 6.1 msec, and the amplitude was 533 ± 201 μV. Bf-M3 and Rf-Late responses were not observed in the control subjects (Table 1) (Fig. 1).

**Table 1:** Electrophysiological parameters of normal subjects and spastic groups

	Normal	Hemiparetic		Paraparetic	Spasticity		
	Left side	Unaffected side	Affected side	Severe side	Total	p*	p**
T lat(ms)	17.9 ± 1.9	18.0 ± 1.8	17.3 ± 2.5	18.1 ± 2.1	17.7 ± 2.3	0.853	0.389
T amp (μV)	6404 ± 2859	3038 ± 1943	6579 ± 4369	7356 ± 4092	7146 ± 4126	0.636	0.000
Bf-M2 Lat (ms)	33.6 ± 6.1 (24 %)	26.1 ± 10.1 (12.5 %)	33.0 ± 12.8 (35 %)	39.6 ± 10.7 (50 %)	36.6 ± 11.9(41 %)	0.009	0.058
Bf-M2 Amp (μV)	533 ± 201	394 ± 464	389 ± 262	408 ± 320	421 ± 290	0.006	0.582
Bf-M3 lat (ms)	NR	78.5 ± 0.7 (8 %)	64.7 ± 25.7 (53 %)	87.9 ± 40.8 (60 %)	77.4 ± 35.5(55 %)	-	0.360
Bf-M3 Amp (μV)	NR	271 ± 323	373 ± 241	299 ± 175	335 ± 209	-	0.019
Accelerometer(ms)	51.4 ± 9.8	55.2 ± 8.9	54.5 ± 9.6	49.5 ± 8.9	51.8 ± 9.4	0.659	0.820

*Normal and Spasticity **All groups

*Pc* was found to be 4.8 ± 1.5 (beat range; 2–10). Ɵ _*1*_ was found to be 18.3 ± 5.6°; *t* was 317.4 ± 23.4 msec. The mean *ω*_*1*_ was 58.0 ± 16.6°/s, and *f* was 1.0 ± 0.1 Hz (range; 0.8–2.0). The *DR* was calculated as 0.6 ± 0.2 (range; 0.2–0.9) (Table 2).

**Table 2:** Kinesiological parameters of normal subjects and spastic groups

	Normal	Hemiparetic		Paraparetic	Spasticity		
	Left side	Unaffected side	Affected side	Severe side	Total	p*	p**
Pc	4.8 ± 1.5	3.1 ± 1.6	4.6 ± 2.6	4.8 ± 1.6	4.7 ± 2.1	0.734	0.006
Ɵ_1_(°)	18.3 ± 5.6	11.6 ± 6.9	13.7 ± 6.6	16.2 ± 6.9	14.8 ± 6.8	0.061	0.001
t1(msec)	317.4 ± 23.4	294.7 ± 54.5	250.1 ± 80.8	261.7 ± 79.9	255.5 ± 80.0	0.001	0.001
ω1(°/s)	58.0 ± 16.6	40.7 ± 19.5	56.2 ± 24.5	65.4 ± 31.2	60.5 ± 28.0	0.537	0.004
f (Hz)	1.0 ± 0.1	1.0 ± 0.1	1.4 ± 0.6	1.2 ± 0.4	1.3 ± 0.5	0.000	0.000
Ɵ_2_(°)	10.6 ± 5.6	4.2 ± 3.7	7.2 ± 5.1	8.6 ± 5.3	7.8 ± 5.2	0.044	0.001
ω2(°/s)	41.7 ± 23.2	20.7 ± 16.1	31.5 ± 21.2	40.3 ± 21.6	35.6 ± 21.7	0.415	0.001
DR	0.6 ± 0.2	0.3 ± 0.3	0.4 ± 0.3	0.5 ± 0.2	0.47 ± 0.2	0.187	0.01
n	25	24	35	30	65	N/S G	

*Normal and Spasticity **All groups

### Electrophysiological and kinesiological features of the spasticity group

**Fig. 2:**
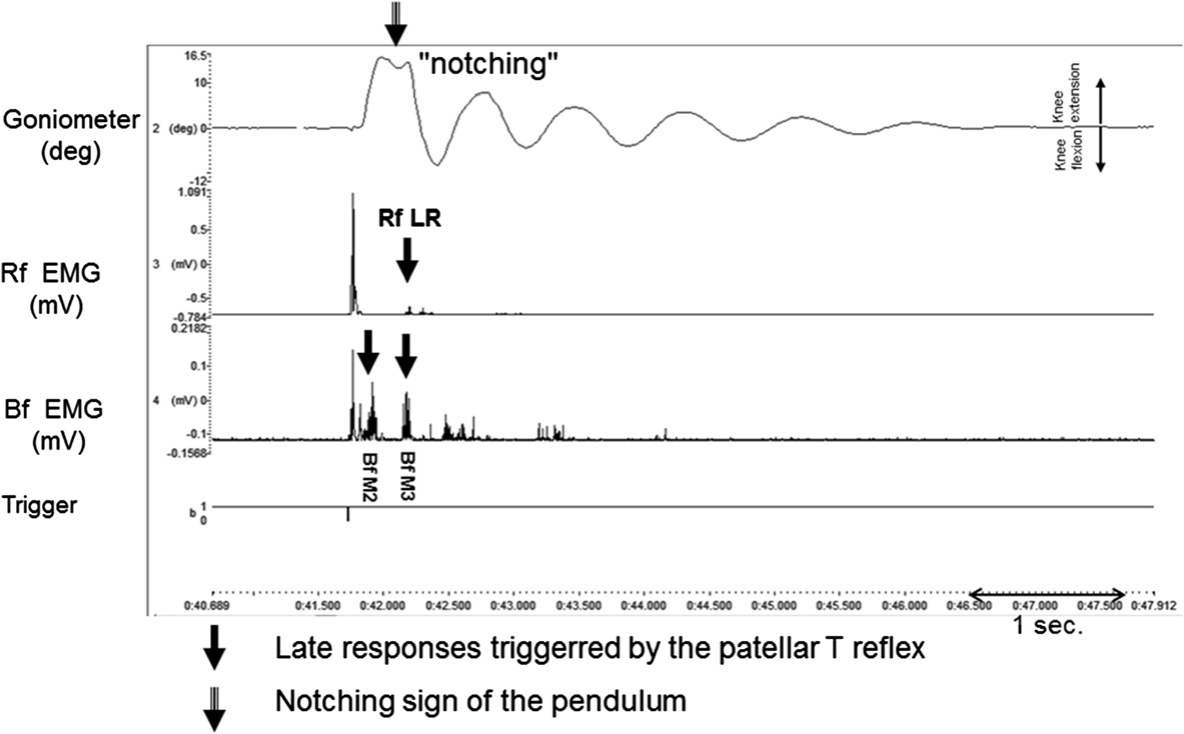
Ashworth Grade 3 spastisicity in a hemiplegic case / R, Affected side. The top trace was recorded from an Ashworth 3 spastic patient, using a goniometer. The “notching” sign shown in the trace is not observed in normal subjects. In the second trace, the late responses in the rectus femoris and biceps femoris muscles following the patellar T reflex are observed. In the third trace, the temporal relationship between the “notch” sign and the medium and late latency reflex responses is noted. The pendulum ends with a five beat (Pc) oscillation. EMG recordings have been rectified. The bottom trace indicates that the patellar T reflex has been triggered. The scale is shown on the left vertical line. The upper trace is the angle in degrees, while the middle and lower traces are shown in mV amplitude. The sweep speed is shown on the bottom right

**Fig. 3:**
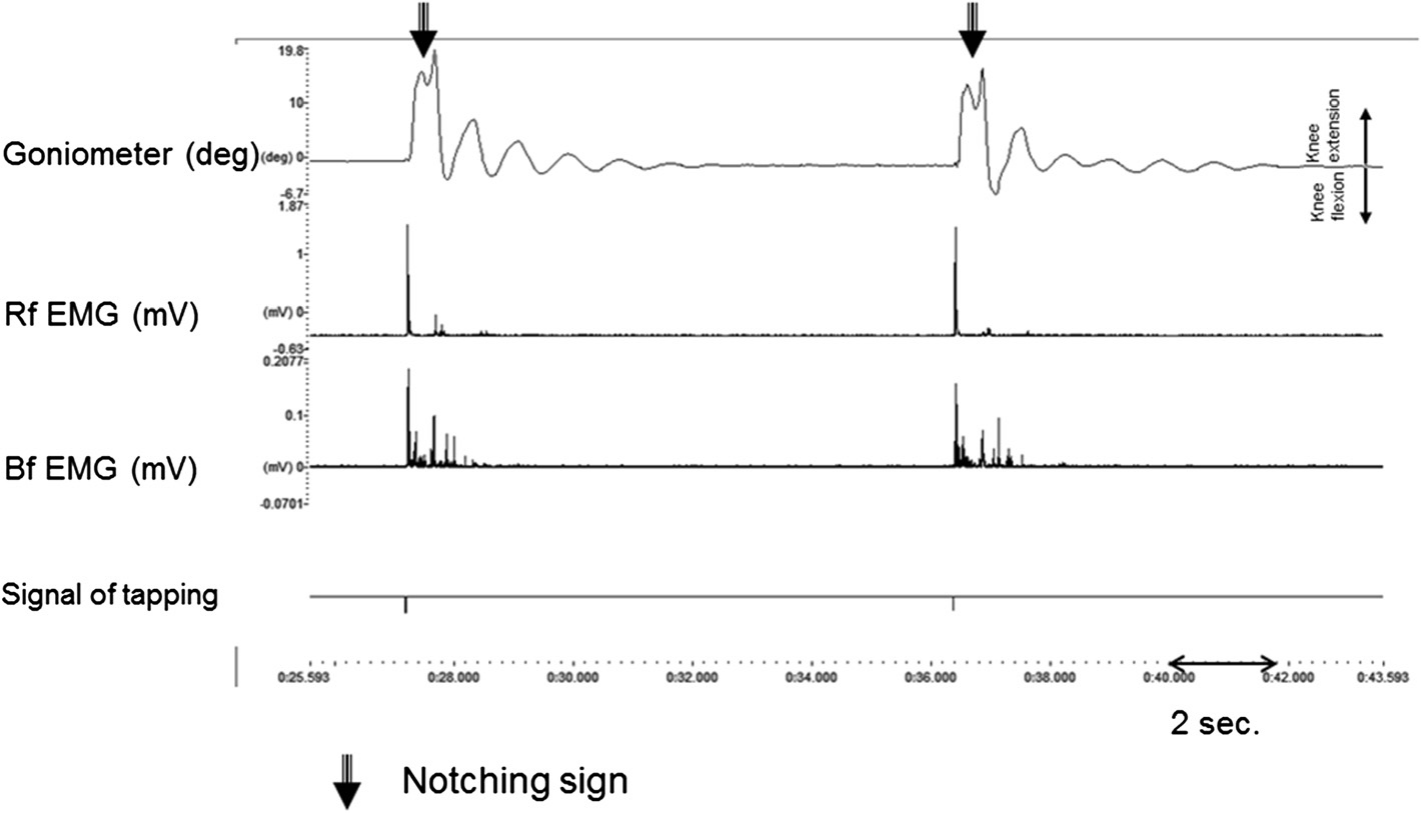
Ashworth Grade 2 spastisicity in a paraplegic case. “Notching” signs seen in the Ashworth 2 spastic paraplegic patient are observed in two consecutive pendulums. In the second trace, late responses observed in the rectus femoris muscle are unrelated to the subsequent beat of the oscillation. In the third trace, intensive Bf-M2 and M3 reflex responses emerged in the biceps femoris muscle. EMG recordings have been rectified. The bottom trace shows signs of triggering the patellar T reflex. The scale is shown on the left vertical line. The upper trace is the angle in degrees, while the middle and lower traces are shown in mV amplitude. The sweep speed is shown on the bottom right

The patellar T reflex was obtained in each case. The latency of the T reflex was 17.7 ± 2.3 msec, and the amplitude was 6938.1 ± 4228.9 μV, similar to the results of the control subjects. Bf-M2/M3 responses were observed in 54 patients (84 %) (Figs. 2 and 3), while the corrected latency of Bf-M2 was measured as 36.6 ± 11.9 msec, and the amplitude was 398.5 ± 325.9 μV. The latency of the corrected Bf-M3 was found to be 77.4 ± 35.5 msec, and the amplitude was 335.6 ± 209.5 μV. LLR in the Rf muscle were observed in 20 % of the patients. The beginning time of the joint movement was measured as 51.9 ± 9.4 msec using an accelerometer (Table 3).

**Table 3:** Electrophysiological parameters of normal subjects and patients in spastic group according to the Ashworth scale

	Normal	Spasticity	Ash 1	Ash 2	Ash 3	Ash 4	p*	p**
T lat(ms)	17.9 ± 1.9	17.7 ± 2.3	17.4 ± 2.3	17.8 ± 2.1	18.0 ± 2.4	16.5 ± 3.1	0.853	0.164
T amp (μV)	6404 ± 2859	7146 ± 4126	5315 ± 2832	8738 ± 4758	8344 ± 2623	683 ± 338	0.636	0.000
Bf-M2 Lat(ms)	33.6 ± 6.1(24 %)	36.6 ± 11.9(41 %)	34.5 ± 13.8	40.2 ± 9.6	39.3.7 ± 6.7	23.9.7 ± 9.4	0.175	0.104
Bf-M2 Amp (μV)	533 ± 201	421 ± 290	400 ± 325	507 ± 340	345 ± 171	173 ± 98	0.215	0.003
Bf-M3 lat (ms)	NR	77.4 ± 35.5(55 %)	77.7 ± 52.2	69.9 ± 26.6	83.9 ± 26.6	101 ± 25	-	0.600
Bf-M3 Amp (μV)	NR	335 ± 209	322 ± 161	393 ± 268	293 ± 149	149.5 ± 47	-	0.251
Accelerometer(ms)	51.4 ± 9.8	51.8 ± 9.4	48.7 ± 8.6	55.9 ± 9.1	50.3 ± 10	54.2 ± 10	0.659	0.346

*Normal and Spasticity **Ashworth groups

**Fig. 4:**
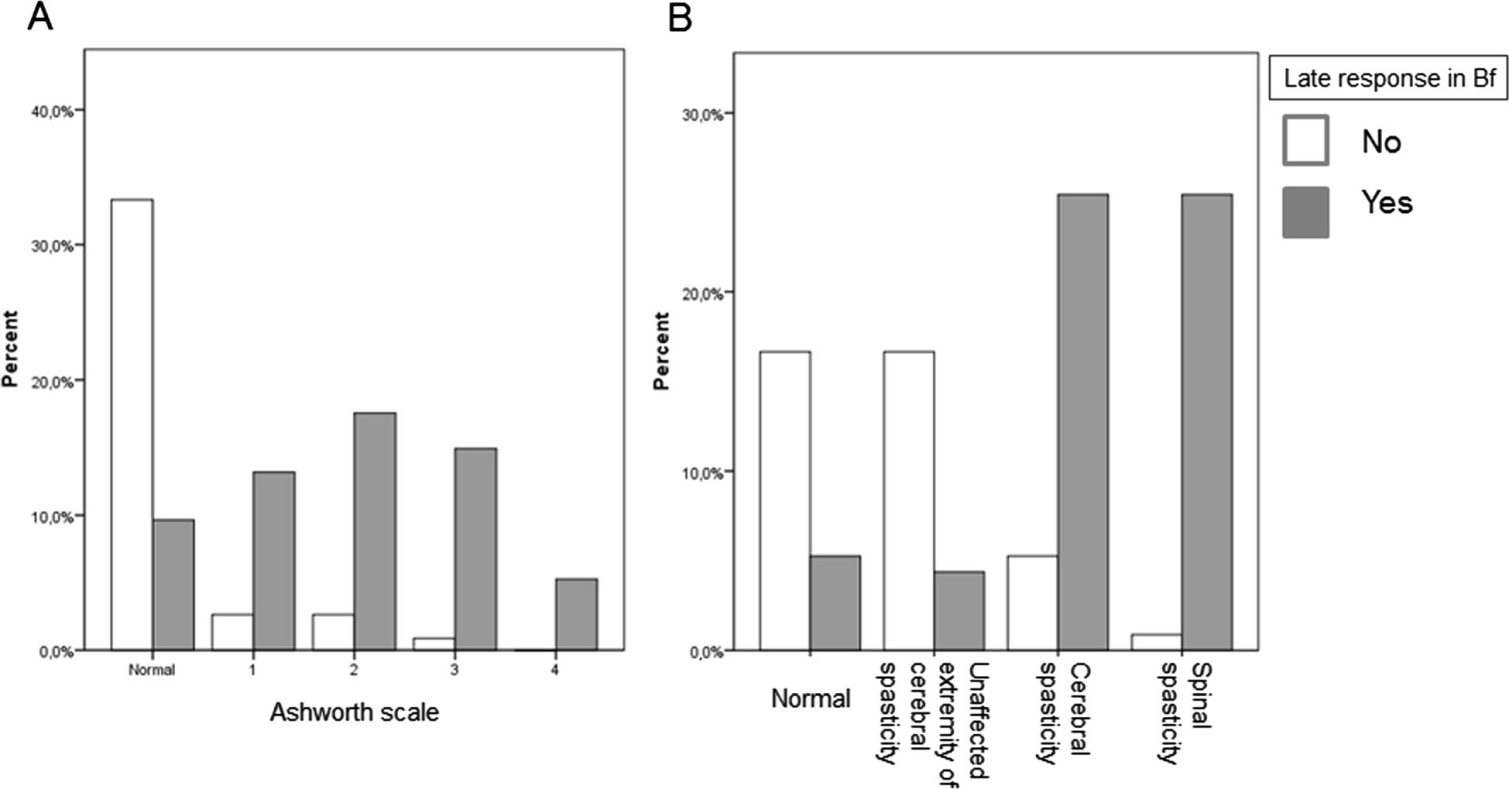
Late responses observed in the biceps femoris muscle according to the Ashworth scale (**a**) and distribution within groups (**b**), late responses increase significantly with spasticity

The *Pc* (count of the pendulum) was found to be 4.7 ± 2.2 in the spasticity group. Ɵ_*1*_ was found to be 14.8 ± 6.8°; the extension time was 255.5 ± 80.0 msec, *ω*_*1*_ was 60.5 ± 28.0°/s, and *f* was 1.3 ± 0.5 Hz (range; 0.9–4.5). The *DR* was calculated as 0.5 ± 0.3 (range; 0–0.9) (Table 2).

The *Pc, t, Ɵ*_*2*_*, ω*_*2*_ and *DR* decreased as the scale increased. The *Pc* was found to be 7 (6–10 min-max) in the Ashworth 1 group, 4.9 (3.3–7 min-max) in the Ashworth 2 group and 3.3 (1–6 min-max) in the Ashworth 3 group, while it was 1 in the Ashworth 4 group. The Ɵ_*1*_ was similar in groups 1 and 2, while the Ashworth 4 group had the smallest angle. The highest *ω*_*1*_ was found in the third group, followed by the second and first groups, while the lowest angular velocity was observed in the Ashworth 4 group. The frequency increased along with increasing spasticity, consistent with the scale. The most prominent change compatible with the AS was observed in the *DR* (0.74, 0.53, 0.28, and 0, respectively) (Table 4).

**Table 4:** Kinesiological parameters of normal subjects and patients in spastic group according to the Ashworth scale

	Normal	Spasticity	Ash 1	Ash 2	Ash 3	Ash 4	p*	p**
Pc	4.8 ± 1.5	4.7 ± 2.1	7.0 ± 1.0	5.0 ± 1.0	3.0 ± 2.0	1.0 ± 0.0	0.734	0.000
Ɵ_1_(°)	18.3 ± 5.6	14.8 ± 6.8	16.9 ± 6.6	17.1 ± 5.2	13.7 ± 5.6	3.1 ± 3.4	0.061	0.001
t1(msec)	317.4 ± 23.4	255.5 ± 80.0	304 ± 55	279 ± 56	208 ± 82	162 ± 74	0.001	0.000
ω1(°/s)	58.0 ± 16.6	60.5 ± 28.0	58 ± 25	64 ± 23	70.5 ± 29	23 ± 25	0.537	0.016
f (Hz)	1.0 ± 0.1	1.3 ± 0.5	1.1 ± 0.2	1.1 ± 0.2	1.5 ± 0.5	2.3 ± 0.8	0.000	0.000
Ɵ_2_(°)	10.6 ± 5.6	7.8 ± 5.2	12.6 ± 4.8	8.9 ± 2.7	4.4 ± 2.9	-	0.044	0.000
ω2(°/s)	41.7 ± 23.2	35.6 ± 21.7	53 ± 21	40 ± 12	25 ± 16	-	0.415	0.000
DR	0.6 ± 0.2	0.47 ± 0.2	0.75 ± 0.1	0.53 ± 0.1	0.28 ± 0.2	0.0 ± 0.0	0.187	0.000
N	25	65	18	23	18	6	N/S G	Ash.

*Normal and Spasticity **Ashworth groups

The amplitude of the patellar T reflex was similar in Ashworth 2 and 3 groups, while the highest value was observed in the Ashworth 2 group. Lower amplitude values were observed in the Ashworth 1 group compared to 2 and 3, but the lowest amplitude value was found in the Ashworth 4 group. The Bf-M2 response was lowest in the Ashworth 1 group (72.2 %), while this value was found to be 91.3 % in the Ashworth 2 group. The Bf-M2 response was observed in all of the subjects in the Ashworth 3 and 4 groups Fig. 4. The Bf-M2 amplitude was highest in the Ashworth 2 group followed by groups 1 and 3, while the lowest value was obtained in the Ashworth 4 group. The highest Bf-M3 response was found in the Ashworth 3 group (94.4 %), followed by the Ashworth 2 and Ashworth 1 groups (90.4 and 61.1 %), while the lowest rate was observed in the Ashworth 4 group (33.3 %). Continuous after-discharges were monitored following the stretch responses in all 6 patients in the Ashworth 4 group.

**Fig. 5:**
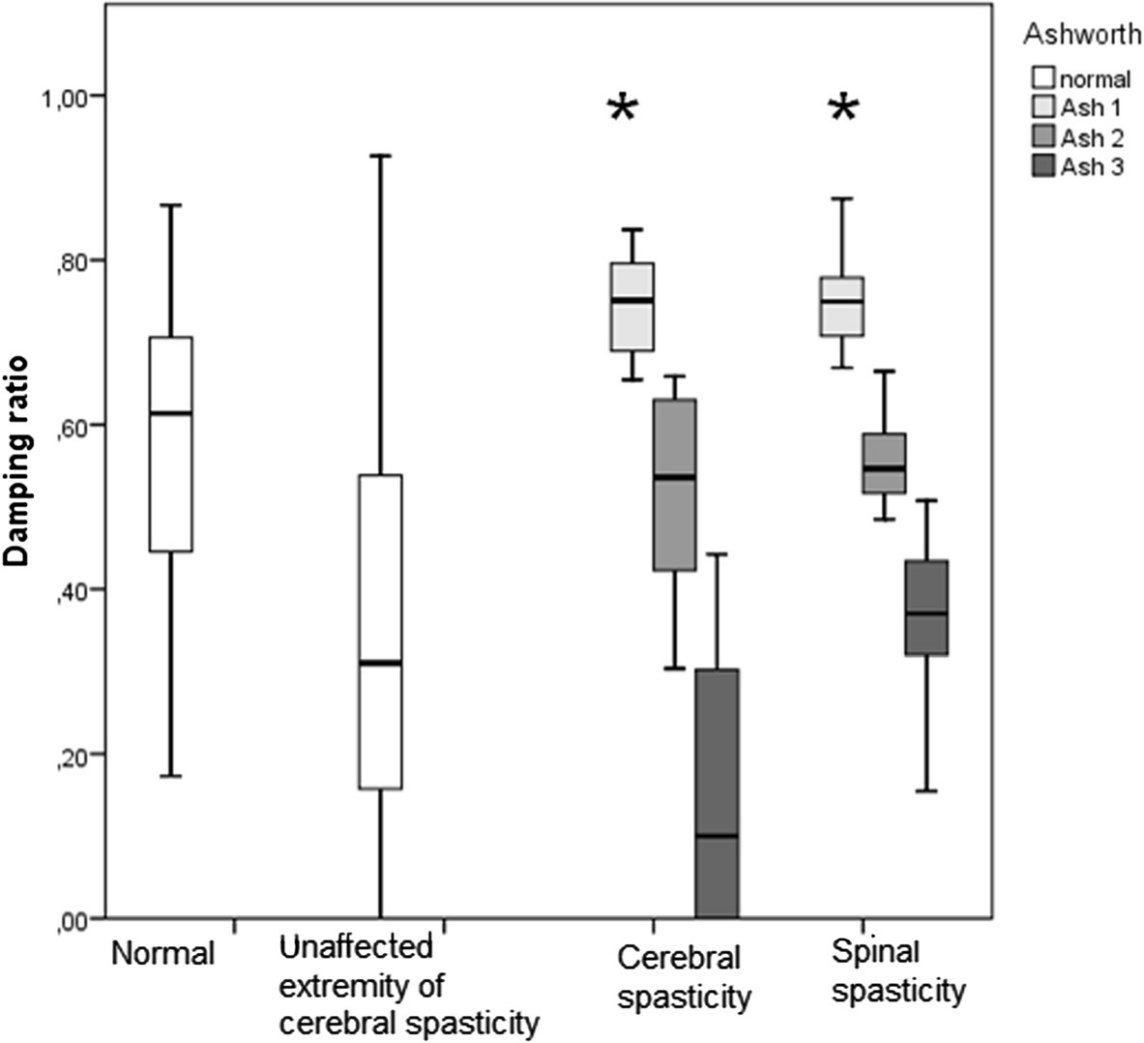
Relationship between damping ratio and grade of spasticity in different type lesions. The damping ratio is distributed to be compatible with the Ashworth scale. The distribution of the damping ratio of normal subjects also includes the values of the spastic patients. However, in the non-paretic limbs of hemiplegic patients the damping ratio distribution coincides closely with that of the spastic group

Regarding comparisons carried out according to the AS, significant correlations were found in all of the parameters except *ω*_*1*_. The AS showed a very strong negative correlation with the *DR* (Fig. 5) (Spersman’s rho correlation, Correlation coefficient −0.919, *p* = 0.000) and a strong negative correlation with the *Pc* (Spersman’s rho correlation, Correlation coefficient −0.842, *p* = 0.000). No correlation was found between the electrophysiological parameters and the spasticity group according to the AS.

### Electrophysiological and kinesiological features of the cerebral and spinal spasticity subgroups

When spasticity patients were compared in terms of cerebral and spinal etiologies, no differences were found between the Ashworth 1 and 2 cases in terms of kinesiological parameters. With regard to electrophysiological parameters, there were differences in the T reflex amplitude and in the latency and amplitude of the Bf-M2 and M3 according to the AS (Table 3) (Fig. 3).

With regard to kinesiological parameters, significant differences were observed only in the Ɵ_*1*_ and Ɵ_*2*_ angles in the Ashworth three patients (Mann–Whitney U, *p* = 0.035 and *p* = 0.030, respectively). Ɵ_*1*_ was found to be 9.5 ± 4° in cerebral spasticity patients and 15.1 ± 6.4° in spinal spasticity patients. Ɵ_*2*_ was found to be 2.1 ± 2.7° in cerebral spasticity patients and 5.3 ± 2.7° in spinal spasticity patients. In other words both of the angles were smaller in the cerebral spasticity group (Table 2).

Because there were five cerebral-originated patients and only one spinal-originated spastic patient in the Ashworth 4 level, no comparison was made and the groups were combined.

### Notching sign of the pendulum

A finding was encountered in the spastic group that was not observed in the control groups. In the control subjects, all oscillations of the pendulum triggered by the patellar T reflex had a symmetric structure in the goniometric recordings (Fig. 1). However, an important structural change was observed in the patients’ oscillation. The pattern observed in the first oscillation of the pendulum triggered by the patellar reflex was noticeable, and we termed this the “notching sign” (Figs. 2 and 3). The notching sign was observed in 67 % of the patient group. “Plateau-like” deformations were seen in the first oscillation of the pendulum in 15 % of the patients, which we did not observe in the control subjects. The structure of the pendulum was impaired in all but 18 % (42 % of Ashworth 1 patients) of patients. This type of deformation was observed in 80 % of the Ashworth 1 patients, 83 % of the Ashworth 2 patients, and 86 % of the Ashworth 3 patients, whereas the notching sign was observed in 62 % of the cerebral-origin patients. The notching sign was found in 36 % of the Ashworth 1, 50 % of the Ashworth 2, and 100 % of the Ashworth 3 subjects, whereas the notching sign was observed in 38 % of the spinal-origin patients. There was pendular deformation in all of the Ashworth 4 cases. Furthermore we did not observe any notching effect without Bf muscle activity.

## Discussion

Medium and long latency discharges are rarely seen in the agonist Rf muscle which is stretched actively at the beginning of the pendulum and antagonist Bf muscle which is stretched passively in control subjects, while these discharges are seen in high rates in the spastic groups. In the majority of cases, after-discharges in the form of a polyphasic complex follow the patellar T reflex response in the Rf muscle and stretch reflex responses in the Bf muscle, and may last about one second in the spastic cases. We observed an increase in time and amplitude of the after-discharges, consistent with the AS. We also observed an increase both in duration and amplitude of the responses seen in the Rf, which were compatible with the patellar clonus, in addition to almost uninterrupted after- discharges with increased amplitude, which were seen in the Bf following stretch reflex responses in the Ashworth 4 group patients. Joint movements were restricted, amplitude and angles of the motion decreased, and the duration shortened as the duration and amplitude of after discharges increased. It has been suggested that these discharges, which begin late, are long, and are severely asynchronous, emerge through polysynaptic tracts. Dimitrijevic et al. reported that easily inducing polysynaptic tracts responsible for after-discharges is a characteristic of spasticity [19, 20].

Among the stretch reflex responses, the second peak seen after the SLR on EMG is known as the medium latency reflex (MLR). The MLR response occurs through a pathway mediated by group II and group Ia muscle spindle afferents [21]. The MLR response also markedly increases in spastic patients. This response emerges after 40–50 msec (range; 19.1–65 msec) in Bf [22]. In this study, latency of the Bf- M2 response was found to be 50 msec. The Bf-M2 response was observed in most of the spastic patients, while it was seen in only one case in the control group. In their study conducted on the wrist flexor carpi radialis muscles in spastic patients, Cody et al. failed to demonstrate a correlation between MLR amplitude and spastic muscle tone, while Berardelli et al. found a correlation between both parameters [23, 24]. In this study, the M2 response was seen at the lowest rate in the Ashworth 1 group and was observed at increased rates as the scale increased. The M2 response was observed in all patients in the Ashworth 3 and 4 groups, supporting a possible correlation between spasticity level and M2 response. The longest latency M2 response was seen in the Ashworth 1 group, while the shortest latency response was observed in the Ashworth 4 group. Likewise, the T reflex and amplitude of M2 was highest in the Ashworth 2 group and lowest in the Ashworth 4 group. Of the stretch responses observed in the Bf muscle, the likelihood of observing M2 and M3 either together or separately prominently increased as the AS level increased, indicating an important associated feature of change in spastic tone.

The AS is associated with resistance by the extremity to passive motion. Therefore, this scale is expected to affect the frequency of the patellar pendulum motion, mean angular velocity of oscillations, number of pendulums, and damping ratio. The *ω*_*1*_ was found to be highest in the spastic cases, while no significant difference was found in the control subjects. Angular velocity was the same in the Ashworth 1 group as in the control subjects, while this value was higher in the Ashworth 2 and Ashworth 3 groups and significantly lower in the Ashworth 4 group. Thus, groups 2 and 3 could easily be distinguished from the control subjects through angular velocity. The low value of *ω*_*1*_ in the Ashworth 4 group indicated that another phenomenon had become dominant. It could be said that antagonist muscle activity, as seen in the EMG recordings, and the mechanical factors caused by the change in intrinsic features of the muscle restrain the angular velocity of joint motion.

The Ɵ_*1*_ value was lower in the spastic cases than in the control subjects. Angle value prominently decreased in the Ashworth 4 group. Joint motion observed in the spasticity group with low AS rating was close to that of the control subjects. It was impaired as the scale increased, and a “notching sign” was observed at the end of the extension before flexion was initiated. Notching and plateauing, which impaired the structure of the pendulum, was found to conform with the M3 reflex muscle activity observed in the antagonist muscle. This suggests that triggered stretch reflex responses and after discharges affect this phenomenon. This was thought to be a counterpart of the “catch” term defined in the Tardieu scale [25].

We also observed that patients with severe spasticity could not complete pendular motion of the joint as expected in normal extension-flexion and extension. In other words, pendular motion terminated after a very short period of flexion following extension in some patients. The period of time preceding extension was shorter in the spasticity patients than in the control subjects. This time was much shorter in the Ashworth 4 group; however, this extension occurred too quickly. Therefore, the factors generating angular velocity create a better understanding of high and low values in angular velocity. There was markedly increased angular velocity in the Ashworth 2 and 3 groups. This could be explained by the participation of the motor units that were rapidly ignited, as well as some motor units recruited with the shortened latency by means of decreases in homosynaptic depression due to spasticity. Consequently, the amplitude of the reflex response was the second factor affecting angular velocity. Because the amplitude shows the number of active motor units and this number shows the contraction power, it is clear that powerful contractions lead to rapid motion, weak contractions are accompanied by slow movement [26].

In terms of mean frequency, the control subjects had the lowest frequency value. Conversely, in the spastic group, the mean frequency value increased in correlation with the AS and reached the maximum value of 2.3 Hz. The high value of frequency in the spastic group can be defined as a feature of muscle stiffness. We accept that a true perception of high frequency during deep tendon reflex examination was a factor in pathological assessment. It could be said that gain change in the tendon reflex afferents through different pathways determines the patellar T reflex and pendulum. The amplitude of the reflex response and mean angular velocity of the first oscillation present in a dichotomic nature in the spasticity group. The frequency of the first pendular oscillation increases in correlation with the AS, while the damping ratio decreases with the scale. Dichotomic features could be explained by changes in the stretch reflex features such as in Bf, an antagonist of the quadriceps femoris muscle, that triggers the pendulum. The ability of this antagonist muscle group to be activated by an increased level of spasticity can change features of the pendulum. Therefore, features of the pendulum determined by the quadriceps femoris muscle, such as the maximal extension angle and duration, tend to increase in the levels in which activity of the antagonist muscle is not determinative. The present tendency is to decrease with the effects of the antagonist muscle caused by the stretch reflex as the spasticity level increases. Remarkably, an increase in the incidence of Bf-M2 and Bf-M3 demonstrated a correlation with the AS. We consider that the activation of the antagonist muscles at a certain level of spasticity creates this phenomenon and could be evaluated as a determinant of spasticity.

The AS presents a correlation with the kinesiological parameters, demonstrating the reason why we still use this scale despite strong opposing arguments and evidence [2, 27]. It is possible that the problem resulted in distinguishing the control subjects and spastic cases in terms of kinesiological parameters. Electrophysiological parameters observed in this study and also specified in the other studies could be defined as a primary tool in distinguishing the control subjects and spastic cases [9]. We concluded that late responses in the Bf muscle and after discharges observed in the Rf muscle are important in the diagnosis of spasticity. In addition, the “notching sign” observed in the first oscillation of the pendulum is an indicator of spasticity.

Spasticy is as an adaptive/maladaptive response to a corticospinal tract lesion, with changes to muscle structure and function due to that inactivity. A more comprehensive clinical method of testing neural and non-neural contributions to impairments and function is needed. Kinetic gait analysis showed congruent increase of the mechanical resistance to joint rotation in children with cerebral palsy [28] . The pathophysiological profile of spasticty does not only consist of dynamic spasticity, but it results from the simultaneous contribution of paresis, co-contraction, immature, and non-neural components [28]. The Ashworth scale cannot differentiate spasticity from contracture in children with cerebral palsy, however the Tardieu Scale can provide this information [29]. Consequently, the triggered stretch reflex responses and after-discharges affect the notching phenomenon, as a counterpart of the Tardieu’s catch phenomenon, which have a potential to improve spasticy assessment.

## Conclusion

Late responses in the Bf and Rf muscles provide important insights into the muscles’ responses to passive stretch after a motor system lesion. The kinesiological characteristics of spasticity are in agreement with the AS, showing that this scale is indispensable at present. We hope that this approach will contribute not only to the assessment of spasticity at rest but also during voluntary movements [30]. Finally, whether the clinical usefulness of kinesiologic and electrophysiologic appraisal is superior to clinical assessment, as the Ashworth Scale correlates well with kinesiological data, along with its potential to offer quantitative measurements remain to be explored.

## Abbreviations

(AS): Ashworth scale
(Bf): Biceps femoris muscle
(*DR*): Damping ratio
(EMG): Electromyography
(*f*): Frequency
(MLR) (synonym M2): Medium latency reflex
(LLR)(synonym M3): Late latency response
(SLR)(synonym M1): Short latency response
*(Ɵ)*: Maximum extension angle
*(Ɵ1)*: Maximum extension angle in the first oscillation
*(Ɵ2)*: Maximum extension angle in the second oscillation
(Rf): Rectus femoris muscles
(*t*_*1*_): Time for maximal extension in the first oscillation
(*t*_*2*_): Time for maximal extension in the second oscillation
(*ω*_*1*_): Mean angular velocities in the first oscillation
(*ω*_*2*_): Mean angular velocities in the second oscillation
